# Serial Complete Blood Count-Derived Inflammatory Indices in Acute Pancreatitis: Temporal Associations with Disease Severity, Pancreatic Necrosis, and Prolonged Hospitalization—A Retrospective Single-Center Study

**DOI:** 10.3390/jcm15218304

**Published:** 2026-10-25

**Authors:** Şaheste Şen, Engin Altıntaş, Ebru Çetin Kenan, Burcu Bozkurt Burgucu

**Affiliations:** 1 Department of Internal Medicine, Orhangazi State Hospital, Bursa 16800, Türkiye; 2 Department of Internal Medicine, Faculty of Medicine, Mersin University, Mersin 33343, Türkiye; 3 Department of Emergency Medicine, Bursa City Hospital, Bursa 16250, Türkiye; 4 Department of Gastroenterology, Medicana Bursa Hospital, Bursa 16110, Türkiye

**Keywords:** acute pancreatitis, neutrophil-to-lymphocyte ratio, platelet-to-lymphocyte ratio, systemic immune-inflammation index, pancreatic necrosis, prognosis, inflammation

## Abstract

**Background:** Acute pancreatitis (AP) evolves during the first days of hospitalization, and the prognostic value of complete blood count (CBC)-derived inflammatory indices may depend on measurement timing. We evaluated serial neutrophil-to-lymphocyte ratio (NLR), platelet-to-lymphocyte ratio (PLR), and systemic immune-inflammation index (SII) values and their associations with disease severity, pancreatic necrosis, and prolonged hospitalization. **Methods:** This retrospective single-center study included 299 adults hospitalized with AP between 2010 and 2020. NLR, PLR, and SII were assessed at admission, 24 h, and 48 h. Temporal changes and between-group differences were analyzed, and discriminatory performance was evaluated using receiver operating characteristic analysis. **Results:** NLR, PLR, and SII decreased over time in the overall cohort but remained higher in patients with moderate-to-severe AP. Admission PLR had the highest observed AUC for moderate-to-severe AP (0.738). Admission values did not significantly distinguish patients with pancreatic necrosis, whereas 48-h SII had the highest observed AUC for necrosis (0.810; sensitivity 82.35%; specificity 76.95%). For hospitalization > 4 days, 48-h NLR had the highest observed AUC (0.742). **Conclusions:** Observed discriminatory performance varied according to both measurement timing and clinical outcome. Later measurements showed greater numerical discrimination for pancreatic necrosis and prolonged hospitalization than admission values. These findings are exploratory, and the proposed cut-offs require external validation.

## 1. Introduction

Acute pancreatitis (AP) ranges from a mild, self-limited illness to disease complicated by local complications, organ failure, intensive care admission, and death [1,2,3,4]. The revised Atlanta classification defines severity according to local or systemic complications and the presence and duration of organ failure [4].

Diagnosis is based on the combination of compatible symptoms, pancreatic enzyme elevation, and imaging when indicated [5,6]. Etiology and subsequent clinical course are heterogeneous; recurrent disease and later pancreatic complications occur in a subset of patients, and the distribution of common causes such as gallstones and alcohol varies among populations [7,8].

Early risk stratification is therefore a routine part of AP management. Hemoconcentration and serum creatinine have been studied as early markers of organ failure or pancreatic necrosis [9,10], and several multivariable scores, including APACHE II, Ranson, BISAP, and SIRS-based approaches, are used to estimate severity [11,12]. These scores are clinically useful, but their performance and practical requirements differ, and some depend on multiple variables or observation over time [13,14,15,16]. This leaves room for simple markers that can be derived from tests already obtained in routine care.

Complete blood count (CBC)-derived inflammatory indices are attractive in this regard because they require no additional sampling or laboratory testing. The neutrophil-to-lymphocyte ratio (NLR) has been associated with AP severity and adverse outcomes, although reported thresholds and diagnostic performance vary substantially across studies [17,18,19,20,21,22].

Other CBC-derived measures have also been studied. White blood cell count and related hematological parameters vary with the clinical course of AP [23]. The platelet-to-lymphocyte ratio (PLR) has been linked to disease severity and adverse outcomes [24,25], and leukocyte-derived indices have been explored for the early identification of necrotizing disease [26].

These associations are biologically plausible because neutrophils, lymphocytes, and platelets participate in the systemic inflammatory response to pancreatic injury. Neutrophil activation contributes to both pancreatic and systemic injury [27], while red cell and leukocyte-derived measures have been associated with disease severity [28,29,30]. The systemic immune-inflammation index (SII), which combines neutrophil, platelet, and lymphocyte counts, has likewise been associated with AP severity and clinically relevant outcomes [31,32,33,34].

Timing may be as important as the marker itself. AP can evolve over the first several days, and current international guidance emphasizes repeated clinical assessment rather than reliance on presentation findings alone [35]. Laboratory and inflammatory parameters also change during this period [36]. Recent studies support this time-dependent view: NLR measured again within 24–48 h retains prognostic value, while other cohorts have examined admission SII together with BISAP or inflammatory indices reassessed at 48 h [37,38,39].

We therefore examined NLR, PLR, and SII at admission, 24 h, and 48 h in patients hospitalized with AP. In addition to disease severity and pancreatic necrosis, hospitalization > 4 days was included as a pragmatic measure of a more prolonged clinical course. This threshold reflected the pre-existing grouping in the original dataset, in which the median length of hospital stay was 4 days. We hypothesized that patients with more severe disease, pancreatic necrosis, or prolonged hospitalization would show higher NLR, PLR, and SII values during the first 48 h, with differences becoming more apparent on serial measurement.

## 2. Materials and Methods

### 2.1. Study Design and Population

This retrospective observational study included adult patients hospitalized with a diagnosis of acute pancreatitis in the Department of Gastroenterology at Mersin University Faculty of Medicine Hospital between 1 January 2010 and 31 December 2020.

The diagnosis of AP was based on the standard diagnostic framework requiring at least two of the following three criteria: characteristic acute abdominal pain, serum amylase and/or lipase levels at least three times the upper limit of normal, or imaging findings consistent with acute pancreatitis [4,5,6].

Patient data were retrospectively retrieved from the hospital electronic medical record system, Nucleus Medical Information System (MONAD Yazılım ve Danışmanlık A.Ş., Ankara, Türkiye; version not recorded). A total of 299 patients who met the study criteria were included in the analysis.

Patients younger than 18 years, those with unavailable laboratory data, patients with chronic pancreatitis or hematological diseases, and patients receiving chemotherapy were excluded.

### 2.2. Data Collection and Laboratory Parameters

Demographic and clinical data, including age, sex, APACHE II score, length of hospitalization, intensive care unit (ICU) admission, pancreatic necrosis, and in-hospital outcome, were obtained from the electronic medical records.

CBC parameters obtained at hospital admission (0 h), 24 h, and 48 h were evaluated. These included hemoglobin, hematocrit, WBC count, neutrophil count, lymphocyte count, monocyte count, platelet count, and red cell distribution width (RDW).

CBC-derived inflammatory indices were calculated at each time point using the following formulas:

NLR = neutrophil count/lymphocyte count

PLR = platelet count/lymphocyte count

SII = (neutrophil count × platelet count)/lymphocyte count

where NLR denotes the neutrophil-to-lymphocyte ratio, PLR the platelet-to-lymphocyte ratio, and SII the systemic immune-inflammation index.

### 2.3. Clinical Outcomes and Patient Grouping

Disease severity was analyzed according to the categories available in the original dataset. Although the Revised Atlanta Classification defines mild, moderately severe, and severe AP, the original dataset recorded severity as mild versus a combined moderate-to-severe category and did not separately code the latter two categories. We therefore retained this two-group classification rather than attempting a post hoc three-tier reclassification.

Patients were also categorized according to the presence or absence of pancreatic necrosis. The presence of pancreatic necrosis was determined from the clinical and imaging information recorded in the original dataset.

Length of hospitalization was also evaluated as a clinical outcome. The original dataset grouped hospital stay as ≤4 days and >4 days, and the median length of stay in the cohort was 4 days. We therefore retained >4 days as a pragmatic, cohort-specific definition of prolonged hospitalization rather than as a universally validated clinical threshold.

The principal analyses focused on serial NLR, PLR, and SII measurements and their associations with disease severity, pancreatic necrosis, and prolonged hospitalization. Measurements obtained at admission, 24 h, and 48 h were evaluated to determine whether discriminatory performance differed according to the timing of measurement.

### 2.4. Statistical Analysis

Continuous variables were summarized using mean ± standard deviation (SD) and, where appropriate, median and interquartile range (IQR). Categorical variables were expressed as numbers and percentages. The original statistical plan used parametric methods based on the overall cohort size and the central limit theorem.

Comparisons of continuous variables between two independent groups were performed using Student’s *t*-test. Changes in laboratory parameters and inflammatory indices across admission, 24 h, and 48 h were evaluated using repeated-measures analysis of variance (ANOVA). Pairwise comparisons between measurement time points were performed using contrast analysis.

Receiver operating characteristic (ROC) curve analysis was performed to assess the discriminatory ability of NLR, PLR, and SII for disease severity, pancreatic necrosis, and prolonged hospitalization. Areas under the curve (AUCs) with 95% confidence intervals (CIs), cut-off values, sensitivity, and specificity were reported from the original ROC analyses.

Statistical analyses were performed using IBM SPSS Statistics for Windows, version 21.0 (IBM Corp., Armonk, NY, USA), and MedCalc Demo software (MedCalc Software Ltd., Ostend, Belgium; exact version number was not recorded in the original study documentation). A two-sided *p* value < 0.05 was considered statistically significant.

## 3. Results

### 3.1. Baseline Characteristics and Clinical Outcomes

A total of 299 patients with AP were included in the study. The mean age was 54.2 ± 17.3 years, and 149 patients (49.8%) were male. Most patients were classified as having mild AP (*n* = 241, 80.6%), whereas 58 patients (19.4%) were included in the moderate-to-severe AP group. The mean APACHE II score was 5.6 ± 3.4, with a median of 5 [IQR, 4–8].

The median length of hospitalization was 4 days [IQR, 3–6]. During hospitalization, 16 patients (5.4%) required intensive care, pancreatic necrosis was identified in 17 patients (5.7%), and two patients (0.7%) died. Baseline characteristics and key clinical outcomes of the study population are summarized in Table 1.

### 3.2. Temporal Changes in Cbc-Derived Inflammatory Markers

WBC and neutrophil counts decreased significantly during the first 48 h of hospitalization, from 12.15 ± 4.96 and 9.52 ± 4.78 at admission to 9.89 ± 4.75 and 7.15 ± 4.59 at 48 h, respectively (both *p* < 0.001). Platelet counts also decreased significantly over time (*p* < 0.001), whereas lymphocyte counts showed a significant overall temporal change (*p* = 0.005), with higher values at 48 h than at 24 h.

The CBC-derived inflammatory indices followed a similar pattern. Mean NLR decreased from 8.71 ± 8.03 at admission to 6.99 ± 5.99 at 24 h and 5.30 ± 5.35 at 48 h (*p* < 0.001). PLR decreased from 217.38 ± 148.73 to 181.28 ± 119.59 and 152.19 ± 97.61, respectively (*p* < 0.001). Similarly, SII decreased from 2205.65 ± 2201.42 at admission to 1652.49 ± 1725.77 at 24 h and 1162.49 ± 1371.05 at 48 h (*p* < 0.001). Temporal changes in the selected CBC-derived inflammatory markers are summarized in Table 2.

Complete temporal changes in all evaluated CBC parameters are provided in Supplementary Table S1.

### 3.3. Inflammatory Indices According to Clinical Outcomes

Patients in the moderate-to-severe AP group had significantly higher NLR, PLR, and SII values than those with mild AP at all three time points (all *p* < 0.001). At admission, mean NLR was 14.16 ± 10.95 versus 7.66 ± 6.80, PLR was 325.05 ± 188.19 versus 196.31 ± 129.04, and SII was 3764.99 ± 2929.61 versus 1895.90 ± 1863.53 in the moderate-to-severe and mild AP groups, respectively. These differences remained significant at both 24 and 48 h.

In contrast, admission NLR, PLR, and SII did not differ significantly according to the presence of pancreatic necrosis. Differences became apparent during follow-up. At 24 h, patients with pancreatic necrosis had higher NLR (12.35 ± 7.50 vs. 6.67 ± 5.74; *p* < 0.001), PLR (253.46 ± 112.57 vs. 176.93 ± 118.79; *p* = 0.010), and SII (3405.87 ± 2908.54 vs. 1546.79 ± 1574.67; *p* < 0.001). At 48 h, NLR and SII remained significantly higher in patients with necrosis (*p* = 0.001 for both), whereas the difference in PLR was no longer statistically significant (*p* = 0.073).

A similar pattern was observed for length of hospitalization. Admission NLR and PLR did not differ significantly between patients hospitalized for ≤4 days and those hospitalized for >4 days (*p* = 0.125 and *p* = 0.266, respectively), whereas admission SII was higher in patients with prolonged hospitalization (2566.39 ± 2490.51 vs. 1995.70 ± 1991.26; *p* = 0.030). At both 24 and 48 h, NLR, PLR, and SII were significantly higher in patients hospitalized for >4 days than in those hospitalized for ≤4 days (all *p* < 0.001). NLR, PLR, and SII values according to disease severity, pancreatic necrosis, and length of hospitalization are summarized in Table 3.

### 3.4. Discriminative Performance of Inflammatory Indices

ROC analysis showed moderate discrimination of the three inflammatory indices for the moderate-to-severe AP group. Admission PLR had the highest AUC (0.738, 95% CI 0.684–0.788), followed closely by admission SII (AUC 0.737, 95% CI 0.683–0.787) and 24 h NLR (AUC 0.730, 95% CI 0.675–0.780). At a cut-off value of >202.65, admission PLR had a sensitivity of 78.0% and a specificity of 63.07%.

For pancreatic necrosis, the highest observed AUC was for SII measured at 48 h (0.810, 95% CI 0.761–0.853). At a cut-off value of >1359.88, 48-h SII had a sensitivity of 82.35% and a specificity of 76.95%. The 24-h SII AUC was 0.804, while the AUC for 48-h NLR was 0.781.

For hospitalization > 4 days, 48-h NLR had the highest observed AUC (0.742, 95% CI 0.688–0.790), with a sensitivity of 60.91% and a specificity of 80.42% at a cut-off value of >5.21. The corresponding AUC for 48-h SII was 0.719, whereas the highest observed PLR AUC was 0.618 at 24 h. The ROC parameters with the highest observed AUCs for each clinical outcome are summarized in Table 4.

## 4. Discussion

The observed differences in CBC-derived indices varied by both clinical outcome and measurement time. NLR, PLR, and SII declined during the first 48 h in the cohort as a whole, yet remained higher in patients with moderate-to-severe AP. Pancreatic necrosis showed a different pattern: the groups were similar at admission, and separation emerged during follow-up, particularly for NLR and SII.

This time-dependent behavior is relevant to early AP assessment. Established scores remain useful but differ in complexity, variables required, and the time needed for calculation [13,14,15,16,40,41]. Current guidance also recommends repeated reassessment during the early course of illness [35]. CBC-derived indices cannot replace these approaches, but they can be calculated repeatedly from tests that are already part of routine care.

For NLR, values were higher in the moderate-to-severe group at all three time points, with the highest AUC at 24 h (0.730). The corresponding cut-off of >6.56 had 70.0% sensitivity and 71.37% specificity. Previous studies have also linked higher NLR with severe AP and adverse outcomes, although the reported thresholds differ widely [17,18,19,20,21,22]. Differences in case mix, severity definitions, sampling time, and chosen endpoints are likely to explain part of this variation. The threshold identified here should therefore be viewed as specific to this cohort.

PLR was also higher in the moderate-to-severe group throughout the first 48 h, with its highest AUC at admission (0.738). Associations between elevated PLR and AP severity have been reported in individual studies and, more recently, in meta-analytic data [24,25,42,43]. Across the outcomes examined here, however, the highest observed AUC was not consistently associated with the same index, arguing against reliance on a single CBC-derived marker for all clinical outcomes.

SII showed a similar association with disease severity. Liu et al. reported an AUC of 0.920 for severe AP [31], Biyik et al. reported an AUC of 0.809 [32], and Salehi et al. found discrimination of approximately 0.74 for separating mild from moderate-to-severe disease [33]. More recent work has also linked SII with clinically relevant outcomes in AP [34]. The spread of these estimates is notable and probably reflects differences in populations, endpoints, and timing of measurement rather than a single reproducible cut-off.

The serial design of the present analysis is important because inflammatory markers do not remain fixed after admission. Dancu et al. documented changes in laboratory and inflammatory parameters during follow-up, including at 48 h [36], and dynamic assessment of the modified Endothelial Activation and Stress Index at 48 h has also been associated with adverse outcomes [44]. Although these studies used different markers, they support the practical value of looking at trajectories rather than only baseline values.

Recent 2026 studies make this comparison especially relevant. A meta-analysis showed that NLR remains prognostically informative when reassessed within the first 48 h [37]. Damar and Yaman reported that admission SII added prognostic information to BISAP for severe AP and mortality [38], while Wang et al. found stronger discrimination for AP severity when several inflammatory indices were reassessed at 48 h [39]. Our study addresses a somewhat different question: NLR, PLR, and SII were measured in parallel at admission, 24 h, and 48 h and compared across three clinical outcomes. The clearest timing effect was seen for pancreatic necrosis, for which admission values showed little separation but follow-up values did.

Among patients with pancreatic necrosis, all three indices were higher at 24 h, and NLR and SII remained higher at 48 h. SII had the highest observed AUC, measuring 0.804 at 24 h and 0.810 at 48 h. At the 48-h cut-off of >1359.88, sensitivity was 82.35% and specificity was 76.95%; the 48-h NLR AUC was 0.781.

The lack of a clear difference at admission, followed by separation at 24–48 h, is noteworthy and may reflect persistence of the inflammatory response in patients who develop pancreatic necrosis. However, only 17 patients had necrosis, and AUCs across time points were not formally compared. The ROC estimates and derived thresholds should therefore be regarded as exploratory rather than evidence that later testing is definitively superior.

For prolonged hospitalization, the pattern was similar but less specific. NLR, PLR, and SII were higher at both 24 and 48 h in patients hospitalized for >4 days, whereas admission NLR and PLR were not different between groups. The highest observed AUC was for 48-h NLR (0.742; cut-off >5.21; sensitivity 60.91%; specificity 80.42%), followed by 48-h SII (AUC 0.719).

Hospital length of stay is influenced by more than inflammatory burden. Comorbidity, local complications, treatment decisions, institutional practice, and discharge criteria all contribute. These indices should therefore be interpreted as correlates of a more prolonged clinical course rather than as determinants of length of stay.

WBC count also changed significantly over time. Leukocytosis has long been associated with AP severity, but its prognostic performance is inconsistent [23,26]. A recent cohort of 172 patients likewise identified WBC among the laboratory variables associated with severe AP [45]. In our data, WBC was less discriminative than the main CBC-derived indices and is better viewed as a complementary marker.

NLR, PLR, and SII capture overlapping but not identical components of the inflammatory response: NLR reflects the balance between neutrophilia and lymphopenia, PLR incorporates platelet changes, and SII combines all three cell lines [27,28,29,30]. Their ease of calculation has also led to study in other acute-care settings. For example, hematological indices including NLR varied across T-MACS risk categories in non-ST-elevation acute coronary syndrome, although their added prognostic value beyond the clinical score was limited [46]. This wider use supports their practicality but also underlines that they are nonspecific markers of systemic inflammation.

The retrospective, single-center setting also limits how broadly these findings can be interpreted. Local referral patterns, treatment practices, and data-recording procedures may have influenced both serial laboratory measurements and clinical outcomes. The observed associations should therefore be interpreted within the context of this cohort and generalized cautiously to other clinical settings.

### Limitations

The study has several limitations. It was retrospective and single-center, which introduces the possibility of selection and information bias and limits generalizability. In addition, the 2010–2020 study period may have introduced temporal variability, as changes in acute pancreatitis management, imaging practices, and laboratory procedures over time could have influenced patient assessment and outcome ascertainment. Pancreatic necrosis occurred in only 17 patients, and only two patients died, limiting reliable event-specific analyses and the precision of the ROC estimates for necrosis. The derived cut-off values should therefore be regarded as exploratory, cohort-specific thresholds rather than clinically validated decision limits. Because the ROC-derived cut-off values were generated and evaluated within the same cohort, their performance may be optimistic and external validation is required. AUCs were not formally compared across markers or time points, and numerical differences should not be interpreted as evidence of statistical superiority. The number of comparisons across markers, outcomes, and time points also increases the possibility of chance findings. The analyses were primarily unadjusted; therefore, the observed associations do not establish that NLR, PLR, or SII provide independent prognostic information beyond established clinical predictors or severity scores. Potential confounding from treatment-related factors, including fluid resuscitation volumes and baseline medication use, could not be accounted for. The original statistical plan selected parametric methods based on the overall cohort size and the central limit theorem; distributional diagnostics were not documented in the original statistical analysis, which is particularly relevant when interpreting comparisons involving the small necrosis subgroup. These indices can also be affected by hydration status, infection, comorbidity, and the interval from symptom onset to sampling. Finally, the original dataset combined moderately severe and severe AP, preventing analysis of the full three-tier Revised Atlanta Classification.

Within these constraints, the study provides a three-time-point view of routinely available inflammatory indices during the first 48 h of AP. The highest observed AUC varied by outcome and time point: admission PLR for the combined moderate-to-severe group, 48-h SII for pancreatic necrosis, and 48-h NLR for hospitalization >4 days. These numerical differences should be viewed as hypothesis-generating because formal comparisons between AUCs were not performed.

## 5. Conclusions

Serial CBC-derived inflammatory indices were associated with disease severity, pancreatic necrosis, and prolonged hospitalization, but the highest observed AUC differed by outcome and time point. Admission PLR had the highest observed AUC for the combined moderate-to-severe group, whereas 48 h SII had the highest observed AUC for pancreatic necrosis and 48 h NLR for hospitalization > 4 days. The emergence of necrosis-related differences at 24–48 h, rather than at admission, is the main temporal finding of this study. Because the analysis was retrospective, largely univariable, and included only 17 patients with pancreatic necrosis, the proposed cut-offs should be considered exploratory until they are externally validated. These associations were unadjusted and should not be interpreted as demonstrating independent prognostic value beyond established clinical predictors. Prospective, multicenter studies are needed to confirm these associations and to determine whether serial CBC-derived inflammatory indices provide reproducible clinical value across different patient populations.

## Figures and Tables

**Table 1:** Baseline characteristics and key clinical outcomes of the study population (*n* = 299).

Characteristic	Value
Age, years	54.2 ± 17.3
Median age [IQR], years	53 [40–68]
**Sex, *n* (%)**	
Male	149 (49.8)
Female	150 (50.2)
**Disease severity, *n* (%)**	
Mild AP	241 (80.6)
Moderate-to-severe AP *	58 (19.4)
APACHE II score	5.6 ± 3.4
Median APACHE II [IQR]	5 [4–8]
Length of hospital stay, days	5.1 ± 3.8
Median length of stay [IQR], days	4 [3–6]
ICU admission, *n* (%)	16 (5.4)
Pancreatic necrosis, *n* (%)	17 (5.7)
In-hospital mortality, *n* (%)	2 (0.7)

Values are presented as mean ± standard deviation, median [interquartile range], or *n* (%), as appropriate. AP, acute pancreatitis; APACHE II, Acute Physiology and Chronic Health Evaluation II; ICU, intensive care unit; IQR, interquartile range. * The moderate-to-severe AP category represents the combined severity group available in the original dataset; moderately severe and severe AP were not separately coded.

**Table 2:** Temporal changes in selected CBC-derived inflammatory markers in the overall study population.

Marker	Admission	24 h	48 h	*p* for Time
WBC	12.15 ± 4.96	10.91 ± 4.97 ^a^	9.89 ± 4.75 ^a^	<0.001
Neutrophils	9.52 ± 4.78	8.40 ± 4.86 ^a^	7.15 ± 4.59 ^a^	<0.001
Lymphocytes	1.65 ± 1.08	1.59 ± 0.74	1.74 ± 0.77 ^b^	0.005
Platelets	259.85 ± 97.06	231.87 ± 91.26 ^a^	222.64 ± 83.88 ^a^	<0.001
NLR	8.71 ± 8.03	6.99 ± 5.99 ^a^	5.30 ± 5.35 ^a^	<0.001
PLR	217.38 ± 148.73	181.28 ± 119.59 ^a^	152.19 ± 97.61 ^a^	<0.001
SII	2205.65 ± 2201.42	1652.49 ± 1725.77 ^a^	1162.49 ± 1371.05 ^a^	<0.001

Values are presented as mean ± standard deviation. WBC, neutrophil, lymphocyte, and platelet counts are expressed as ×10^3^/mm^3^. *p* values represent the overall effect of time obtained using repeated-measures analysis of variance. ^a^ Significantly different from admission; ^b^ significantly different from the 24 h measurement (contrast analysis, *p* < 0.05). NLR, neutrophil-to-lymphocyte ratio; PLR, platelet-to-lymphocyte ratio; SII, systemic immune-inflammation index; WBC, white blood cell count.

**Table 3:** NLR, PLR, and SII values at admission, 24 h, and 48 h according to disease severity, pancreatic necrosis, and length of hospitalization.

Outcome	Marker	Time Point	Reference Group	Higher-Risk Group	*p*
**Disease severity**			**Mild AP**	Moderate-to-severe AP *	
	NLR	Admission	7.66 ± 6.80	14.16 ± 10.95	<0.001
		24 h	5.97 ± 5.04	11.43 ± 7.75	<0.001
		48 h	4.42 ± 3.65	8.80 ± 8.60	<0.001
	PLR	Admission	196.31 ± 129.04	325.05 ± 188.19	<0.001
		24 h	164.40 ± 105.53	246.10 ± 136.10	<0.001
		48 h	139.21 ± 76.29	195.10 ± 114.78	<0.001
	SII	Admission	1895.90 ± 1863.53	3764.99 ± 2929.61	<0.001
		24 h	1401.90 ± 1526.45	2717.92 ± 2111.13	<0.001
		48 h	967.22 ± 918.84	1915.40 ± 2265.94	<0.001
**Pancreatic necrosis**			**Absent**	**Present**	
	NLR	Admission	8.66 ± 8.12	9.51 ± 6.48	0.671
		24 h	6.67 ± 5.74	12.35 ± 7.50	<0.001
		48 h	5.06 ± 5.29	9.30 ± 4.79	0.001
	PLR	Admission	215.19 ± 149.26	253.76 ± 138.73	0.300
		24 h	176.93 ± 118.79	253.46 ± 112.57	0.010
		48 h	149.70 ± 97.72	193.46 ± 88.33	0.073
	SII	Admission	2165.92 ± 2209.23	2864.79 ± 2013.67	0.204
		24 h	1546.79 ± 1574.67	3405.87 ± 2908.54	<0.001
		48 h	1096.41 ± 1330.20	2258.75 ± 1606.67	0.001
**Length of hospitalization**			**≤4 days**	**>4 days**	
	NLR	Admission	8.16 ± 7.96	9.64 ± 8.11	0.125
		24 h	5.42 ± 4.50	9.69 ± 7.18	<0.001
		48 h	3.69 ± 2.78	8.07 ± 7.25	<0.001
	PLR	Admission	210.08 ± 145.84	229.94 ± 153.44	0.266
		24 h	160.44 ± 96.94	217.08 ± 144.39	<0.001
		48 h	134.73 ± 65.68	182.17 ± 131.04	<0.001
	SII	Admission	1995.70 ± 1991.26	2566.39 ± 2490.51	0.030
		24 h	1237.48 ± 1171.37	2365.55 ± 2228.03	<0.001
		48 h	792.98 ± 590.63	1797.39 ± 1973.31	<0.001

Values are presented as mean ± standard deviation. *p* values represent between-group comparisons at each time point using Student’s *t*-test. AP, acute pancreatitis; NLR, neutrophil-to-lymphocyte ratio; PLR, platelet-to-lymphocyte ratio; SII, systemic immune-inflammation index. * The moderate-to-severe AP category represents the combined severity group available in the original dataset; moderately severe and severe AP were not separately coded.

**Table 4:** ROC parameters with the highest observed AUCs for NLR, PLR, and SII according to disease severity, pancreatic necrosis, and prolonged hospitalization.

Outcome	Marker	Time Point	Cut-Off	AUC (95% CI)	Sensitivity, % (95% CI)	Specificity, % (95% CI)	*p*
Moderate-to-severe AP *	NLR	24 h	>6.56	0.730 (0.675–0.780)	70.00 (55.4–82.1)	71.37 (65.2–77.0)	<0.001
	PLR	Admission	>202.65	0.738 (0.684–0.788)	78.00 (64.0–88.5)	63.07 (56.6–69.2)	<0.001
	SII	Admission	>2362.77	0.737 (0.683–0.787)	68.00 (53.3–80.5)	75.10 (69.1–80.4)	<0.001
**Pancreatic necrosis**	NLR	48 h	>5.31	0.781 (0.730–0.827)	88.24 (63.6–98.5)	69.15 (63.4–74.5)	<0.001
	PLR	24 h	>170.67	0.758 (0.706–0.806)	88.24 (63.6–98.5)	60.99 (55.0–66.7)	<0.001
	SII	48 h	>1359.88	0.810 (0.761–0.853)	82.35 (56.6–96.2)	76.95 (71.6–81.7)	<0.001
**Hospitalization > 4 days**	NLR	48 h	>5.21	0.742 (0.688–0.790)	60.91 (51.1–70.1)	80.42 (74.0–85.8)	<0.001
	PLR	24 h	>134.11	0.618 (0.560–0.673)	70.00 (60.5–78.4)	49.74 (42.4–57.1)	0.0004
	SII	48 h	>1027.30	0.719 (0.665–0.770)	60.91 (51.1–70.1)	77.78 (71.2–83.5)	<0.001

For each marker and clinical outcome, the time point with the highest AUC among the available ROC analyses is presented. AUCs were not formally compared between markers or time points; therefore, ‘highest observed AUC’ refers to numerical differences only. Complete ROC results are provided in Supplementary Table S2. ROC, receiver operating characteristic; AUC, area under the curve; CI, confidence interval; AP, acute pancreatitis; NLR, neutrophil-to-lymphocyte ratio; PLR, platelet-to-lymphocyte ratio; SII, systemic immune-inflammation index. * The moderate-to-severe AP category represents the combined severity group available in the original dataset; moderately severe and severe AP were not separately coded.

## Data Availability

The data are not publicly available due to patient privacy and institutional restrictions.

## Supplementary Materials

The following supporting information can be downloaded at https://www.mdpi.com/article/10.3390/jcm15218304/s1: Table S1: Temporal changes in complete blood count parameters and CBC-derived inflammatory indices in the overall study population. Table S2: Available ROC analyses of NLR, PLR, and SII for disease severity, pancreatic necrosis, and prolonged hospitalization.
