# Supplementary material for: Serial Complete Blood Count-Derived Inflammatory Indices in Acute Pancreatitis: Temporal Associations with Disease Severity, Pancreatic Necrosis, and Prolonged Hospitalization—A Retrospective Single-Center Study
Source: J Clin Med. 2026 Oct 25;15(21):8304. doi: 10.3390/jcm15218304 (PMC13763683; doi:10.3390/jcm15218304)
Supplement: Supplementary file 1 [file jcm-15-08304-s001.zip › jcm-4620581-supplementary.pdf]

Supplementary Materials for: Serial Complete Blood Count-Derived Inflammatory Indices in Acute Pancreatitis: Temporal Associations with Disease Severity, Pancreatic Necrosis, and Prolonged Hospitalization

Supplementary Table S1

Table S1. Temporal changes in complete blood count parameters and CBC-derived inflammatory indices in the overall study population.

Parameter	Admission	24 h	48 h	P for time
Hemoglobin	13.41 ± 2.01	12.44 ± 2.07[a]	12.17 ± 1.92[a]	<0.001
Hematocrit	39.42 ± 5.13	36.95 ± 5.44[a]	36.19 ± 5.01[a]	<0.001
WBC	12.15 ± 4.96	10.91 ± 4.97[a]	9.89 ± 4.75[a]	<0.001
Lymphocytes	1.65 ± 1.08	1.59 ± 0.74	1.74 ± 0.77[b]	0.005
Monocytes	0.78 ± 0.40	0.75 ± 0.42	0.76 ± 0.37	0.403
Neutrophils	9.52 ± 4.78	8.40 ± 4.86[a]	7.15 ± 4.59[a]	<0.001
Platelets	259.85 ± 97.06	231.87 ± 91.26[a]	222.64 ± 83.88[a]	<0.001
RDW	14.49 ± 1.96	14.49 ± 1.88	14.45 ± 1.90	0.410
NLR	8.71 ± 8.03	6.99 ± 5.99[a]	5.30 ± 5.35[a]	<0.001
PLR	217.38 ± 148.73	181.28 ± 119.59[a]	152.19 ± 97.61[a]	<0.001
SII	2205.65 ± 2201.42	1652.49 ± 1725.77[a]	1162.49 ± 1371.05[a]	<0.001

Values are presented as mean ± standard deviation. P values represent the overall effect of time obtained using repeated-measures analysis of variance. [a] Significantly different from admission; [b] significantly different from the 24-h measurement (contrast analysis, $p < 0.05$). Hemoglobin is expressed as g/dL; hematocrit and RDW as %; WBC, neutrophil, lymphocyte, monocyte, and platelet counts as $\times 10^3/\mu L$. NLR, neutrophil-to-lymphocyte ratio; PLR, platelet-to-lymphocyte ratio; RDW, red cell distribution width; SII, systemic immune-inflammation index; WBC, white blood cell count.

Supplementary Table S2

Table S2. Available ROC analyses of NLR, PLR, and SII for disease severity, pancreatic necrosis, and prolonged hospitalization.

**Outcome**	**Marker**	**Time**	**Cut-off**	**AUC (95% CI)**	**Sensitivity, %**	**Specificity, %**	**p**
**Moderate-to-severe AP***	NLR	Admission	>10.44	0.718 (0.663–0.769)	60.00	75.93	<0.001
	NLR	24 h	>6.56	0.730 (0.675–0.780)	70.00	71.37	<0.001
	NLR	48 h	>7.47	0.695 (0.639–0.747)	48.00	84.65	<0.001
	PLR	Admission	>202.65	0.738 (0.684–0.788)	78.00	63.07	<0.001
	PLR	24 h	>184.62	0.697 (0.641–0.750)	62.00	71.78	<0.001
	PLR	48 h	>137.53	0.681 (0.625–0.735)	70.00	62.24	<0.001
	SII	Admission	>2362.77	0.737 (0.683–0.787)	68.00	75.10	<0.001
	SII	24 h	>1563.00	0.724 (0.669–0.774)	74.00	72.20	<0.001
	SII	48 h	>1005.65	0.681 (0.624–0.734)	64.00	68.88	<0.001
**Pancreatic necrosis**	NLR	24 h	>9.11	0.763 (0.710–0.810)	64.71	75.89	<0.001
	NLR	48 h	>5.31	0.781 (0.730–0.827)	88.24	69.15	<0.001
	PLR	24 h	>170.67	0.758 (0.706–0.806)	88.24	60.99	<0.001
	SII	24 h	>1614.57	0.804 (0.754–0.847)	88.24	68.79	<0.001

Outcome	Marker	Time	Cut-off	AUC (95% CI)	Sensitivity, %	Specificity, %	p
	SII	48 h	>1359.88	0.810 (0.761–0.853)	82.35	76.95	<0.001
**Hospitalization >4 days**	NLR	Admission	>7.40	0.576 (0.518–0.632)	52.73	63.49	0.029
	NLR	24 h	>6.56	0.695 (0.640–0.747)	60.00	77.25	<0.001
	NLR	48 h	>5.21	0.742 (0.688–0.790)	60.91	80.42	<0.001
	PLR	Admission	>209.40	0.548 (0.489–0.605)	48.18	65.08	0.169
	PLR	24 h	>134.11	0.618 (0.560–0.673)	70.00	49.74	<0.001
	PLR	48 h	>126.11	0.615 (0.557–0.670)	64.55	56.08	<0.001
	SII	Admission	>2000.25	0.572 (0.513–0.628)	42.27	68.25	0.042
	SII	24 h	>1387.00	0.683 (0.627–0.736)	63.64	73.02	<0.001
	SII	48 h	>1027.30	0.719 (0.665–0.770)	60.91	77.78	<0.001

ROC, receiver operating characteristic; AUC, area under the curve; CI, confidence interval; AP, acute pancreatitis; NLR, neutrophil-to-lymphocyte ratio; PLR, platelet-to-lymphocyte ratio; SII, systemic immune-inflammation index. The moderate-to-severe AP category represents the combined severity group available in the original dataset; moderately severe and severe AP were not separately coded.
